# Effects of Occupational Hazards on Job Stress and Mental Health of Factory Workers and Miners: A Propensity Score Analysis

**DOI:** 10.1155/2020/1754897

**Published:** 2020-08-21

**Authors:** Yaoqin Lu, Zhe Zhang, Huan Yan, Baoling Rui, Jiwen Liu

**Affiliations:** ^1^Department of Occupational and Environmental Health, College of Public Health, Xinjiang Medical University, Urumqi, Xinjiang, China 830011; ^2^Department of Science and Education, Wulumuqi Center for Disease Control and Prevention, Urumqi, Xinjiang, China 830026; ^3^Department of Medical Engineering, The First Affiliated Hospital of Xinjiang Medical University, Urumqi, Xinjiang, China 830054; ^4^Department of Nutrition and Food Hygiene, College of Public Health, Xinjiang Medical University, Wulumuqi, Xinjiang, China; ^5^Xinjiang Engineering Technology Research Center for Green Processing of Nature Product Center, Xinjiang Autonomous Academy of Instrumental Analysis, Wulumuqi, Xinjiang, China

## Abstract

This study is to evaluate the effects of different occupational hazards on job stress and mental health of factory workers and miners. A total of 6120 workers from factories and mining enterprises in seven districts and one district of Urumqi were determined using the stratified cluster random sampling method. The Effort-Reward Imbalance (ERI) questionnaire and the Symptom Checklist-90 (SCL-90) were used to evaluate the effects of occupational hazard factors on job stress and mental health of workers. The propensity score analysis was used to control the confounding factors. The occupational hazards affecting job stress of workers were asbestos dust (OR = 1.3, 95% CI: 1.09-1.55), benzene (OR = 1.25, 95% CI: 1.10-1.41), and noise (OR = 1.39, 95% CI: 1.22-1.59). The occupational hazards affecting the mental health of workers were coal dust (OR = 1.19, 95% CI: 1.02-1.38), asbestos dust (OR = 1.58, 95% CI: 1.32-1.92), benzene (OR = 1.28, 95% CI: 1.13-1.47), and noise (OR = 1.23, 95% CI: 1.07-1.42). Different occupational hazards have certain influence on job stress and mental health of factory workers and miners. The enhancements in occupational hazard and risk assessment, occupational health examination, and occupational protection should be taken to relieve job stress and enhance the mental health of factory workers and miners.

## 1. Introduction

Occupational hazards are personal threats and injuries that occur in the process of production and labor [1] and usually classified into six categories: dust, chemical factors, physical factors, radiological factors, biological factors, and other factors [2]. The specific hazards, potential risk factors, harmful factors, and unsafe behavior of occupational groups may cause occupational accidents and even occupational diseases [3–5]. ILO reports that 2.34 million people die per year in work-related accidents or diseases around the world, and approximately one-quarter of all deaths are related to an unhealthy environment [6]. In addition, 160 million people suffer from nonfatal work-related diseases [7]. According to incomplete statistics, there are 200 million workers exposed to various occupational hazards and more than 16 million people are working in toxic and harmful enterprises, involving more than 30 different job types [8, 9]. However, the importance of safety at the workplace was often overlooked in understanding and practicing occupational health and safety [10].

Current studies mainly focus on the relationship between occupational hazards and occupational diseases [11–13]. However, psychosocial changes in the process of occupational exposure usually happen before physiological disorders, such as job stress and psychological health (anxiety disorder and depression) [14–16]. Nevertheless, these psychosocial hazards are often ignored and lack intervention [17, 18]. In addition, excess psychological stress could decline psychological function and cause negative physiological responses, leading to emotional fluctuations and psychological health problems [19].

Job stress refers to people's response under physiological and psychological pressure caused by the imbalance between occupational needs and individual resources, needs, abilities, and knowledge [20]. With the development of society and the increase in life pressure, people bear more and more pressure from society, work, and life. Job stress has been regarded as the crisis and illness in modern society and life. Factory workers and miners were the occupational group with high demands, low reward, and low job control [21], and their working environment was full of dust, chemical factors, physical factors, and biological factors. Therefore, this paper is to evaluate the effects of occupational hazards on the job stress and mental health of factory workers and miners in Urumqi, China, using the propensity score analysis, in order to provide a theoretical basis to develop prevention and control policies.

## 2. Materials and Methods

### 2.1. Participants

This survey was carried out from January to May 2019. Workers with the occupational exposures to coal dust, silica dust, asbestos dust, benzene, lead, noise, and *Brucella* in factories and mines in Urumqi, China, were investigated. A total of 6500 factory workers and miners were initially selected using a cluster sampling method. Participants working at a production department, assembly line operation, quality assessment, and quality control department were included, and administrative personnel and management personnel were excluded. Those with work experience less than one year or taking psychoactive drugs were also excluded. According to the inclusion and exclusion criteria, 6130 participants were included in this survey. The cross-sectional study was conducted by an online questionnaire using a mobile phone. The respondents volunteered to participate in the survey, and the written informed consent was provided. Finally, 6120 questionnaires were collected, and 10 copies of continuous answer questionnaires were excluded.

### 2.2. Research Methods

A self-administered electronic questionnaire was used to investigate job stress and mental health.

#### 2.2.1. General Investigation

The general investigation included sex, age, ethnicity, educational level, professional title, marital status, labor contracts, working years, monthly income, work schedule, working hours per day, and working days per week.

#### 2.2.2. Job Stress Investigation

The Effort-Reward Imbalance (ERI) model was an important model used to evaluate job stress in the field of occupational health, which was developed by Siegrist in 1996 [15], which was on the basis of the imbalance between effort and reward causing a series of changes in physiological, psychological, and cognitive functions and body health. The ERI questionnaire consisted of three sections: effort (*E*, 6 items), reward (*R*, 11 items), and overcommitment (6 items), totaling 23 items. Each item was assigned a score ranging from 1 (highly disagree) to 5 (highly agree) [20] and was attributed the same weight. The index of an effort-reward ratio was ERI = *E*/(*R*∗*C*), where *C* was the ratio of the number of items of *E* to the number of items of *R*. In this paper, *C* = 6/11. The ERI index of >1, 1, and <l indicated a high effort-low reward return, a balanced effort-reward return, and a low effort-high reward return, respectively. The higher the ratio of the ERI, the worse the job stress [22].

#### 2.2.3. Mental Health Investigation

The Symptom Checklist-90 (SCL-90) was widely used in psychiatric outpatient examination of its high authenticity in evaluating various mental health surveys [23, 24]. SCL-90 involved emotions, consciousness, thinking, feeling, interpersonal relationships, habits, diet, sleep, etc. It can independently assess the state of one's body, cognition, emotion, social function, and spirit. The 90 items were divided into 9 dimensions: somatization, obsessive-compulsive symptom, sensitivity of interpersonal relationship, depression, anxiety, hostility, horror, bigotry, and mental diseases. Each of 90 items ranged from 1 to 5. The higher score showed a worse psychological symptom. According to the Chinese norm, the further examination was needed at the total score of 160 higher, any item score of 2 higher, or the number of positive items more than 43 [25].

### 2.3. Quality Control

The online electronic questionnaires were built using the Questionnaire Star Platform. The preexperiment was conducted in order to ensure the humanistic design and logicality in the answering process. With the assistance of the department managers, the informed consent explaining the questionnaire survey's purpose and the voluntary participation principle was set to all volunteers. A two-dimensional code was used to conduct on-site online investigation. Our staff explained the purpose, significance, content, and requirements to ensure the quality and effectiveness of the online investigation. The data of each online questionnaire was valid data based on the technical design. The continuous answer questionnaires were excluded.

### 2.4. Statistical Methods

Statistical analysis was performed using R (version 3.4.4). The balance of confounding factors was assessed by the propensity score analysis. The multiple logistic regression was employed for multivariate analysis. The Mann-Whitney *U* test was used to compare two groups, and the Kruskal-Wallis *H* test was used to compare more groups of nonparametric (nonnormally distributed) data. A chi-squared test was used for the counting data, and multiple linear regression was employed for multivariate analysis. The significance level (*α*) was set at 0.05.

## 3. Results

### 3.1. General Demographic Characteristics of Factory Workers and Miners

Among the 6120 factory workers and miners, 4017 were men (65.64%) and 2103 were women (34.36%). The occupational hazard factors in which workers are involved were coal dust (1446, 23.63%), silica dust (622, 10.16%), asbestos dust (35, 15.28%), benzene (1947, 31.81%), lead (353, 5.77%), noise (4545, 74.26%), and brucellosis (108, 1.76%) (Table 1).

### 3.2. Comparison of Propensity Score Matching in Job Stress

The survey results showed that 2898 (47.35%) of factory workers and miners suffered from job stress and others (3222, 52.65%) were normal. In order to control confounding factors and eliminate the bias of general demographic characteristics, the propensity score matching was used by selecting a total of 14 factors as the matching covariates, including sex, ethnicity, education level, labor contracts, professional title, work schedule, marital status, monthly income, height, weight, age, working years, working hours per day, and working days per week. The caliper value was set at 0.02. Finally, 2498 pairs of positive and control groups were screened out by a 1 : 1 proximity matching method as shown in Table 2.

Multiple logistic regression analysis was conducted to explore the efficiency of PSA. Job stress and general demographic characteristics were set as the dependent variable and independent variables, respectively. Before matching, the differences of sex (*P* < 0.01), ethnicity (*P* = 0.01), education level (*P* < 0.01), labor contracts (*P* < 0.01), work schedule (*P* < 0.01), monthly income (*P* < 0.01), hypertension (*P* < 0.01), working hours per day (*P* < 0.01), and working hours per week (*P* < 0.01) among the positive group and the control group were statistically significant. After matching, the differences of these variables were eliminated (*P* > 0.05) (Table 3).

### 3.3. Exploration of Factors Influencing Job Stress

According to the multiple logistic regression analysis, the risk factors for job stress were the occupational hazard factors of asbestos dust (OR = 1.30, 95% CI: 1.09-1.55), benzene (OR = 1.25, 95% CI: 1.10-1.41), and noise (OR = 1.39, 95% CI: 1.22-1.59) (Table 4).

### 3.4. Comparison of Propensity Score Matching in SCL

The survey results showed that among 6120 of the factory workers and miners, 2342 (38.27%) suffered from mental health problems and 3778 (61.73%) did not. Using PSM as described in Section 3.2, 2152 positive and control groups were screened out. The differences of sex, ethnicity, education level, labor contracts, work schedule, monthly income, hypertension, working hours per day, and working hours per week were eliminated after matching (Table 5).

### 3.5. Exploration of Factors Influencing Mental Health

According to the multiple logistic regression analysis, the risk factors for mental health problems were the occupational hazard factors of coal dust (OR = 1.19, 95% CI: 1.02-1.38), asbestos dust (OR = 1.58, 95% CI: 1.32-1.92), benzene (OR = 1.28, 95% CI: 1.13-1.47), and noise (OR = 1.23, 95% CI: 1.07-1.42) (Table 6).

## 4. Discussion

Job stress has been proven as a risk factor for the adverse physiological function (such as declined strength, stiffened body, and disorders in sense and memory) and psychological reaction (such as distracted attention and reduced working will and desire) [26, 27]. Ahn et al.'s study has also shown that exposure to occupational hazards adversely affects the individuals' health and exacerbated job insecurity [28]. Factory workers and miners belong to a special professional group, who work in a special environment of high temperature, high pressure, darkness, or dust but have low income and social status. Some studies have already proven that people living in harsh environments have a higher risk of developing mental illnesses and the special environments affect the degree of job stress [29–31]. Our research showed that 47.35% and 38.27% of factory workers and miners suffered from job stress and mental health problems, respectively, suggesting that the status of occupational mental health of factory workers and miners should be paid more attention.

Propensity score matching (PSM) is a statistical matching technique that attempts to estimate the effect of a treatment and intervention by accounting for the covariates that predict receiving the treatment. It attempts to reduce the bias due to confounding variables that could be found in an estimate of the treatment effect obtained from simply comparing outcomes among units that received the treatment versus those that did not [32]. This method can adjust covariates more objectively and scientifically and thereby get more intuitive conclusions. In recent research, the main risk factors for job stress involved sex, professional title, working age, working hours, chronic diseases, and so on. However, the relationship between the risk factors and occupational stress or mental health was less reported. Therefore, this study is the first one to use PSM to explore the relationship between occupational hazards and job stress and mental health. Thus, 14 demographic characteristics of factory workers and miners and the control group, such as sex, ethnicity, and educational level, were adjusted to reduce the confounding bias and thereby obtain more objective results.

The results showed that the influences of occupational hazards on the job stress of factory workers and miners were statistically significant, and the risks of job stress increased 1.30 times, 1.25 times, and 1.39 times with exposure to asbestos dust, benzene, and noise, respectively. Coal dust, asbestos dust, benzene, and noise were the risk factors for psychological health problems of factory workers and miners, and the risks of psychological health problems increased 1.19 times, 1.58 times, 1.28 times, and 1.23 times compared with those who had no exposure, respectively. Occupational dust was an important health risk in modern society, and the adverse health impacts of coal mine particulate matter had been well known [33, 34]. Changes in the body's functioning due to occupational dust exposure, such as developing respiratory symptoms and decreased pulmonary functions, could make workers feel more tired at work and thereby increase the possibility of work errors, leading to psychological burden. Some studies have confirmed the negative effects of chemical substance exposure on tests of psychomotor function and short-term memory [35]. Hence, the long-term occupational exposure to benzene would affect body metabolism, making the workers prone to job stress and psychological problems. Noise was one of the commonest occupational hazards of the modern world. Lai and Huang found that exposure to a high-noise environment would cause endocrine disorders, headaches, blood pressure, and fatigue [36]. In addition, hearing and cognitive ability shared the same neurophysiological conduction, and workers needed to pay more attention of message reception and understanding in a noisy working environment.

It reminded us that setting a barrier between workers and occupational hazards, standardizing operation procedures, and providing protection education training were effective measures to reduce the potential harm and adverse health to workers. Moreover, the use of personal protective devices, such as gloves, glasses, aprons, safety footwear, and dust masks, should be paid more attention by both employees and employers. Improvement of occupational health and safety management has a wide range of benefits for companies, such as the reduction of absenteeism and labor accident and the increase in workers' motivation and productivity [37].

The present study has some limitations to be improved upon in future studies. Firstly, there were still continuously repeated answers in the online questionnaire. Secondly, the cross-sectional investigation cannot prove causality between variables, and the relationship between the factors and diseases needs further investigation. Thirdly, the inclusion and exclusion criteria were not sufficient, such as people's family history in terms of mental and behavioral problems. Then, the effect of PSM was not significant in the case of respondents having similar background. Finally, the influence of single occupational hazard on the mental health of workers and miners was preliminarily described qualitatively, while the influence of pollution level and the interaction of two or more occupational hazards on mental health should be verified in the future studies.

## 5. Conclusion

In this study, the effect of occupational hazard exposure on the job stress and mental health of factory workers and miners in Xinjiang, China, was demonstrated. Asbestos dust (OR = 1.30, 95% CI: 1.09-1.55), benzene (OR = 1.25, 95% CI: 1.10-1.41), and noise (OR = 1.39, 95% CI: 1.22-1.59) were the risk factors for job stress. Coal dust (OR = 1.19, 95% CI: 1.02-1.38), asbestos dust (OR = 1.58, 95% CI: 1.32-1.92), benzene (OR = 1.28, 95% CI: 1.13-1.47), and noise (OR = 1.23, 95% CI: 1.07-1.42) were the risk factors for mental health problems. Therefore, the occupational health monitoring of people with occupational exposure to coal dust, asbestos dust, benzene, and noise should be given more importance in the daily health management of factory workers and miners.

## Figures and Tables

**Table 1:** Characteristics of factory workers and miners.

Items	Groups	Case number	Percentage (%)
Sex	Male	4017	65.64
	Female	2103	34.36
Ethnicity	Han	5016	81.96
	Other	1104	18.04
Education level	Junior high school and below	652	10.65
	High school	1227	20.05
	Junior college	2722	44.48
	Bachelor's degree or above	1519	24.82
Labor contracts	Signed	5896	96.34
	Unsigned	224	3.66
Professional title	None	2349	38.38
	Primary	1326	21.67
	Middle	1483	24.23
	Senior	962	15.72
Work schedule	Day shift	3289	53.74
	Night shift	201	3.28
	Shift	1897	31
	Day and night shifts	733	11.98
Marital status	Unmarried	857	14
	Married	4864	79.48
	Divorced	357	5.83
	Widowed	42	0.69
Monthly income (yuan)	<3000	1656	27.06
	~3000	2093	34.2
	~4000	1329	21.72
	~5000	659	10.77
	~6000	205	3.35
	~7000	86	1.41
	~8000	92	1.5
Chronic disease	Diabetes	364	5.95
	Hypertension	1220	19.93
Age (years)	<25	319	5.21
	~25	634	10.36
	~30	790	12.91
	~35	704	11.5
	~40	723	11.81
	~45	2950	48.2
Working years (years)	<5	920	15.03
	~5	831	13.58
	~10	840	13.73
	~15	319	5.21
	~20	695	11.36
	~25	1266	20.69
	~30	1249	20.41
Working hours per day (hours)	≤7	975	15.93
	>7	5145	84.07
Working days per week (days)	≤5	4006	65.46
	>5	2114	34.54
Occupational hazards	Coal dust	1446	23.63
	Silica dust	622	10.16
	Asbestos dust	935	15.28
	Benzene	1947	31.81
	Lead	353	5.77
	Noise	4545	74.26
	Brucellosis	108	1.76

**Table 2:** Participants before and after matching.

Items	Positive group	Control group
Before matching	2898	3222
After matching	2498	2498
Unmatched	400	724

**Table 3:** Comparison of propensity score matching for job stress.

Variable	*β* (95% CI)	Beta	Standard *β*	OR (95% CI)	VIF	*t*	*P* value
Before matching (*n* = 6120, treated = 2898, control = 3222)							
Intercept	-1.77 (-2.55, -0.99)	0.40	—	0.17 (0.08, 0.37)	—	-4.44	<0.001
Sex	-0.33 (-0.45, -0.21)	0.06	-0.32	0.72 (0.64, 0.81)	1.2	-5.47	<0.001
Ethnicity	0.18 (0.04, 0.32)	0.07	0.14	1.20 (1.04, 1.37)	1.04	2.56	0.01
Education level	0.38 (0.31, 0.44)	0.03	0.7	1.46 (1.37, 1.55)	1.21	11.78	<0.001
Labor contracts	-0.51 (-0.82, -0.20)	0.16	-0.19	0.60 (0.44, 0.82)	1.07	-3.2	<0.001
Professional title	-0.01 (-0.06, 0.04)	0.02	-0.03	0.99 (0.94, 1.04)	1.11	-0.47	0.64
Work schedule	0.08 (0.03, 0.13)	0.02	0.19	1.08 (1.03, 1.14)	1.14	3.37	<0.001
Marital status	-0.07 (-0.19, 0.06)	0.06	-0.06	0.93 (0.82, 1.06)	1.31	-1.06	0.29
Monthly income	-0.08 (-0.13, -0.04)	0.02	-0.21	0.92 (0.88, 0.96)	1.16	-3.73	<0.001
Diabetes	0.17 (-0.05, 0.39)	0.11	0.08	1.19 (0.95, 1.48)	1.06	1.49	0.14
Hypertension	0.34 (0.20, 0.48)	0.07	0.27	1.40 (1.23, 1.62)	1.15	4.85	<0.001
Age	0.05 (0.00, 0.11)	0.03	0.17	1.05 (1.00, 1.11)	3.13	1.79	0.07
Working years	0.04 (0.00, 0.08)	0.02	0.17	1.04 (1.00, 1.08)	2.98	1.81	0.07
Working hours per day	0.28 (0.12, 0.43)	0.08	0.20	1.32 (1.13, 1.54)	1.22	3.52	<0.001
Working days per week	0.22 (0.10, 0.33)	0.06	0.21	1.25 (1.11, 1.39)	1.09	3.75	<0.001
After matching (*n* = 4996, treated = 2498, control = 2498)							
Intercept	-0.35 (-1.22, 0.52)	0.44	—	0.70 (0.30, 1.68)	—	-0.79	0.43
Sex	0.03 (-0.10, 0.16)	0.07	0.03	1.03 (0.90, 1.17)	1.2	0.42	0.67
Ethnicity	0.05 (-0.01, 0.20)	0.08	0.04	1.05 (0.91, 1.22)	1.04	0.67	0.50
Education level	0.02 (-0.05, 0.09)	0.04	0.04	1.02 (0.95, 1.09)	1.22	0.57	0.57
Labor contracts	0.09 (-0.29, 0.47)	0.19	0.03	1.09 (0.75, 1.60)	1.06	0.46	0.65
Professional title	0.01 (-0.04, 0.06)	0.03	0.02	1.01 (0.96, 1.06)	1.1	0.38	0.70
Work schedule	0.01 (-0.04, 0.06)	0.03	0.02	1.01 (0.96, 1.06)	1.13	0.31	0.75
Marital status	-0.02 (-0.16, 0.11)	0.07	-0.02	0.98 (0.85, 1.12)	1.32	-0.32	0.75
Monthly income	-0.01 (-0.06, 0.04)	0.02	-0.02	0.99 (0.95, 1.04)	1.17	-0.37	0.71
Diabetes	0.01 (-0.23, 0.25)	0.12	0.01	1.01 (0.80, 1.29)	1.06	0.1	0.92
Hypertension	0.00 (-0.15, 0.15)	0.08	0	1.00 (0.86, 1.16)	1.15	0.01	0.99
Age	0.01 (-0.05, 0.08)	0.03	0.05	1.01 (0.95, 1.08)	3.27	0.47	0.64
Working years	-0.01 (-0.05, 0.04)	0.02	-0.03	0.99 (0.95, 1.04)	3.06	-0.32	0.75
Working hours per day	0.02 (-0.14, 0.19)	0.09	0.02	1.02 (0.87, 1.21)	1.22	0.26	0.79
Working days per week	0.03 (-0.09, 0.16)	0.06	0.03	1.03 (0.92, 1.17)	1.09	0.54	0.59

**Table 4:** Effects of occupational hazards on the job stress of factory workers and miners according to the multiple logistic regression analysis.

Variable	*β* (95% CI)	Beta	Standard *β*	OR (95% CI)	VIF	*t*	*P* value
Intercept	-0.35 (-0.47, -0.23)	0.06	—	0.70 (0.62, 0.79)	—	-5.75	<0.001
Coal dust	0.04 (-0.10, 0.18)	0.07	0.04	1.04 (0.91, 1.20)	1.15	0.61	0.540
Silica dust	-0.04 (-0.24, 0.16)	0.10	-0.02	0.96 (0.79, 1.18)	1.18	-0.38	0.710
Asbestos dust	0.26 (0.09, 0.44)	0.09	0.19	1.30 (1.09, 1.55)	1.31	2.92	<0.001
Benzene	0.22 (0.09, 0.34)	0.06	0.20	1.25 (1.10, 1.41)	1.12	3.39	<0.001
Lead	-0.24 (-0.50, 0.02)	0.13	-0.11	0.79 (0.61, 1.02)	1.19	-1.83	0.070
Noise	0.33 (0.19, 0.46)	0.07	0.28	1.39 (1.22, 1.59)	1.05	4.83	<0.001
Brucellosis	0.08 (-0.36, 0.52)	0.22	0.02	1.08 (0.70, 1.68)	1.05	0.36	0.720

**Table 5:** Comparison of propensity score matching for mental health.

Variable	*β* (95% CI)	Beta	Standard *β*	OR (95% CI)	VIF	*t*	*P* value
Before matching (*n* = 6120, treated = 2342, control = 3778)							
Intercept	-1.97 (-2.81, -1.13)	0.43	—	0.14 (0.06, 0.32)	—	-4.58	<0.001
Sex	-0.02 (-0.15, 0.10)	0.06	-0.02	0.98 (0.86, 1.10)	1.2	-0.39	0.69
Ethnicity	0.01 (-0.14, 0.15)	0.07	0.01	1.01 (0.87, 1.16)	1.04	0.1	0.92
Education level	0.30 (0.23, 0.37)	0.03	0.57	1.35 (1.26, 1.44)	1.19	8.88	<0.001
Labor contracts	-0.71 (-1.10, -0.32)	0.20	-0.27	0.49 (0.33, 0.73)	1.05	-3.53	<0.001
Professional title	0.05 (0.00, 0.10)	0.03	0.11	1.05 (1.00, 1.10)	1.09	1.89	0.06
Work schedule	0.20 (0.15, 0.25)	0.03	0.48	1.22 (1.16, 1.29)	1.16	7.91	<0.001
Marital status	0.06 (-0.08, 0.19)	0.07	0.05	1.06 (0.93, 1.21)	1.26	0.83	0.4
Monthly income	-0.15 (-0.20, -0.11)	0.02	-0.41	0.86 (0.82, 0.90)	1.16	-6.49	<0.001
Diabetes	-0.14 (-0.37, 0.09)	0.12	-0.07	0.87 (0.69, 1.09)	1.05	-1.21	0.23
Hypertension	0.59 (0.45, 0.73)	0.07	0.49	1.80 (1.57, 2.08)	1.14	8.33	<0.001
Age	0.05 (-0.01, 0.11)	0.03	0.17	1.05 (0.99, 1.12)	3.15	1.59	0.11
Working years	0.13 (0.08, 0.17)	0.02	0.57	1.14 (1.09, 1.19)	3.01	5.68	<0.001
Working hours per day	0.19 (0.03, 0.35)	0.08	0.14	1.21 (1.03, 1.41)	1.22	2.29	0.02
Working days per week	0.10 (-0.01, 0.22)	0.06	0.1	1.11 (0.99, 1.25)	1.08	1.73	0.08
After matching (*n* = 4304, treated = 2152, control = 2152)							
Intercept	-0.47 (-1.49, 0.54)	0.52	—	0.63 (0.23, 1.72)	—	-0.92	0.36
Sex	0.00 (-0.14, 0.14)	0.07	0	1.00 (0.87, 1.15)	1.21	0.04	0.97
Ethnicity	0.07 (-0.09, 0.23)	0.08	0.05	1.07 (0.91, 1.25)	1.05	0.83	0.4
Education level	-0.01 (-0.08, 0.07)	0.04	-0.01	0.99 (0.92, 1.07)	1.21	-0.18	0.86
Labor contracts	0.38 (-0.18, 0.94)	0.28	0.08	1.46 (0.84, 2.56)	1.04	1.34	0.18
Professional title	0.01 (-0.04, 0.07)	0.03	0.03	1.01 (0.96, 1.07)	1.08	0.47	0.64
Work schedule	0.01 (-0.05, 0.07)	0.03	0.02	1.01 (0.96, 1.07)	1.18	0.36	0.72
Marital status	0.04 (-0.10, 0.19)	0.07	0.04	1.04 (0.90, 1.21)	1.23	0.59	0.56
Monthly income	-0.02 (-0.07, 0.04)	0.03	-0.04	0.98 (0.93, 1.04)	1.15	-0.56	0.57
Diabetes	-0.01 (-0.26, 0.23)	0.13	-0.01	0.99 (0.77, 1.26)	1.05	-0.11	0.91
Hypertension	0.09 (-0.06, 0.24)	0.08	0.07	1.09 (0.94, 1.27)	1.13	1.13	0.26
Age	0.01 (-0.06, 0.08)	0.04	0.02	1.01 (0.94, 1.08)	3.17	0.16	0.88
Working years	-0.02 (-0.07, 0.03)	0.03	-0.08	0.98 (0.93, 1.03)	3.07	-0.75	0.46
Working hours per day	0.02 (-0.15, 0.20)	0.09	0.02	1.02 (0.86, 1.22)	1.23	0.28	0.78
Working days per week	-0.02 (-0.15, 0.11)	0.07	-0.02	0.98 (0.86, 1.12)	1.07	-0.28	0.78

**Table 6:** Effects of occupational hazards on the mental health of factory workers and miners according to the multiple logistic regression analysis.

Variable	*β* (95% CI)	Beta	Standard *β*	OR (95% CI)	VIF	*t*	*P* value
Intercept	-0.38 (-0.51, -0.25)	0.07	—	0.68 (0.6, 0.78)	—	-5.69	<0.001
Coal dust	0.17 (0.02, 0.33)	0.08	0.15	1.19 (1.02, 1.38)	1.14	2.22	0.030
Silica dust	0.16 (-0.05, 0.37)	0.11	0.10	1.17 (0.95, 1.45)	1.16	1.47	0.140
Asbestos dust	0.46 (0.28, 0.65)	0.10	0.35	1.58 (1.32, 1.92)	1.27	4.86	<0.001
Benzene	0.25 (0.12, 0.39)	0.07	0.24	1.28 (1.13, 1.47)	1.10	3.75	<0.001
Lead	-0.16 (-0.44, 0.11)	0.14	-0.08	0.85 (0.65, 1.11)	1.16	-1.18	0.240
Noise	0.21 (0.07, 0.35)	0.07	0.18	1.23 (1.07, 1.42)	1.06	2.86	<0.001
Brucellosis	0.29 (-0.17, 0.76)	0.24	0.08	1.34 (0.84, 2.13)	1.04	1.23	0.220

## Data Availability

The data used to support the findings of this study are available from the corresponding author upon request.
